# Severe Acute Respiratory Syndrome Coronavirus-2 Induces Cytokine Storm and Inflammation During Coronavirus Disease 19: Perspectives and Possible Therapeutic Approaches

**DOI:** 10.3389/fphar.2020.592169

**Published:** 2020-11-26

**Authors:** Federica Mannino, Alessandra Bitto, Natasha Irrera

**Affiliations:** Department of Clinical and Experimental Medicine, University of Messina, Messina, Italy

**Keywords:** severe acute respiratory syndrome coronavirus, cytokine storm, inflammation, coronavirus disease, Therapeutics

## Abstract

The new coronavirus outbreak was first identified in Wuhan, China, in December 2019, and has turned out to be a global health emergency, affecting millions of people worldwide. Coronavirus disease 19 (COVID-19), caused by the SARS-CoV-2 virus, can manifest with flu-like symptoms and can be complicated by severe pneumonia with acute respiratory distress syndrome (ARDS); however a large percentage of infected individuals do not have symptoms but contribute to the spread of the disease. Severe acute respiratory syndrome coronavirus-2 infection has become a global public health emergency since no available treatment seems effective and it is hard to manage the several complications caused by an intense release of cytokines. This paper reviews the current options on drugs used to reduce the deadly effects of the cytokine storm.

## Introduction

Every year diseases of viral origin that affect the respiratory tract represent an important problem of public health. The severe acute respiratory syndrome coronavirus (SARS-CoV) in 2002–2003, H1N1 influenza in 2009, and the Middle East respiratory syndrome coronavirus (MERS-CoV) in 2012 significantly increased the rate of mortality among the population worldwide. In March 2020, the WHO declared the coronavirus disease 19 (COVID-19) as a pandemic worldwide that represents a serious public health risk. SARS-CoV, MERS-CoV, and SARS-CoV-2 belong to the coronaviruses class. Coronaviruses are enveloped viruses characterized by a coronary appearance and positive single-stranded RNA genome (Fung and Liu, 2019) that, crossing species barriers, cause illness starting from the “common” cold to more severe diseases.

Patients may show different signs and symptoms that make the differential diagnosis hard for general practitioners; thus the swab sampling remains the crucial point for diagnosis. The most common symptoms include rhinorrhea, fever, nonproductive cough, loss of taste or smell, myalgia, dyspnea, fatigue, and alteration of the leukocyte count (Chakraborty et al., 2020).

Coronaviruses probably originated from bats and then moved to other hosts. In particular, SARS-CoV and MERS-CoV, respectively, infected the Himalayan palm civet and the dromedary camel, before reaching humans, whereas the exact dynamic transmission of SARS-CoV-2 is still unknown, even if the probability of an animal origin is very high, including snakes, birds, marmots, and bats (Chakraborty et al., 2020).

Coronavirus-19 (SARS-CoV-2) is particularly infectious to humans, seriously affects the respiratory tract, and causes the COVID-19 (Du Toit, 2020). The high susceptibility of the population is responsible for the raised incidence of the distribution worldwide so that also COVID-19 has become a serious public health problem.

Coronaviruses, and also SARS-CoV-2, are named “corona” because they have a crown-like appearance if observed under electron microscopy; therefore the term “*coronam,”* the Latin equivalent of “crown,” has been used. In particular, the spike glycoproteins on the envelope of the virus confer the typical shape of a crown (Chan et al., 2013), and both the SARS-CoV-2 pathophysiology and virulence are related to structural and nonstructural proteins (NSPS). NSPS may act by blocking the host innate immune response whereas the structural proteins contribute to viral assembly and release (Lei et al., 2018). The spike glycoproteins of the crown structure are organized in two subunits, S1 and S2, that may link each other creating homotrimers that, in turn, bind the host receptors (Song et al., 2018). The S2 is the highly conserved subunit and, for this reason, represents the target of the antiviral drugs (anti-S2), whereas the spike receptor-binding domain shows a similarity of about 40% to that of the SARS-CoVs. The other structures, such as the open reading frame (ORF) 3b, and the protein encoded by ORF 8 have no homology with the other SARS-CoVs (Cascella et al., 2020).

SARS-CoV-2, as SARS-CoV, specifically binds angiotensin-converting enzyme 2 (ACE2) (Li et al., 2005), using this as the port of entry into the cells. However, SARS-CoV-2 contains some structural differences in the hACE2-binding ridge compared to SARS-CoV, such as the residues 482-485: Gly-Val-Glu-Gly, that increase the binding affinity (Shang et al., 2020).

Therefore, lung damage and inflammation primarily start following the specifying binding to ACE2, but probably it is not the only viral mechanism (Behl et al., 2020; Bordallo et al., 2020; Verdecchia et al., 2020). The exuberant inflammation may certainly be considered as one of the main causes of severity and mortality during SARS-CoV-2 infection.

The aim of this paper is to focus on the possible inflammatory mechanisms mediated by SARS-CoV-2 and the possible therapeutic approaches that might ameliorate the prognosis of the disease, thus reducing the rate of mortality.

## Severe Acute Respiratory Syndrome Coronavirus-2 and Cytokine Storm

SARS-CoV-2 infects the respiratory tract, causing an acute lung injury also characterized by an aggressive inflammatory response (Wong et al., 2004). COVID-19 severity is often related to the exacerbation of the inflammatory process; in fact, high concentrations of cytokines [interleukin (IL)-2, IL-7, IL-10, GCSF, IP10, MCP1, MIP1A, TNFα] were observed in plasma of 13 patients admitted to the intensive care unit (ICU) of Wuhan in China. However, also non-ICU patients (n = 28) showed increased plasma levels of IL-1β, IFN-c, IP-10, MCP-1, IL-4, and IL-10 even if the “cytokine storm” was higher in patients with a severe COVID-19 (Huang et al., 2020).

The increased levels of cytokines and chemokines amplify the innate immune response and stimulate the monocyte‐macrophages, neutrophils, NK cells, and eosinophils recruitment, thus worsening tissue damage related to the acute respiratory distress syndrome: proinflammatory cytokines levels and severity of the disease are strictly linked (Beacon et al., 2020).

Both the excessive immune reaction and the “cytokine storm” caused by the viral infection result in toxicity and respiratory depression up to pulmonary tissue damage which is the leading cause of mortality in COVID-19. One of the main actors of the cytokine storm is IL-6, which, once released by activated leukocytes, stimulates the production of the acute phase proteins, regulates the thermogenesis, and concurs to the cytokine release syndrome characterized by fever and multiple organ dysfunction (Cascella et al., 2020).

Two inflammatory phases may be described during COVID-19: a primary inflammatory response and a secondary inflammatory response. Primary inflammatory response precedes the production of neutralizing antibodies (NAb) and is mainly related to viral replication, viral-mediated ACE2 downregulation, and also the first host response. Secondary inflammatory response occurs when the adaptive immunity is activated and NAb are produced (Fu et al., 2020).

The activation of apoptosis in the epithelial and endothelial cells and pyroptosis in macrophages and lymphocytes leads to the release of proinflammatory cytokines (Yang, 2020).

Previous studies have demonstrated that acute lung injury may be associated with the loss of function of ACE2, which increases vascular permeability and pulmonary edema (Imai et al., 2005). As described, SARS-CoV-2, as SARS-CoV, mediates its entry inside cells through ACE-2 in association with S-protein priming by the host cell protease TMPRSS2; SARS-CoV-2 can downregulate ACE2 and, in turn, could decrease the angiotensin-II clearance, thus worsening tissue damage (Wang et al., 2008; Glowacka et al., 2010; Chatterjee et al., 2020). ACE2 function, in fact, is strictly related to the renin-angiotensin system (RAS): downregulation of ACE2 causes RAS dysfunction, thus increasing inflammation.

The secondary inflammatory response is related to the production of antibodies to face viral infection. The presence of anti-S-neutralizing antibodies in patients highly correlates with the outcome of the disease (Zhang et al., 2006). Fc receptors (FcR) blockade reduces proinflammatory cytokines release but when the virus-anti-S-IgG (anti-spike antibodies) complex binds to the FcR, an inflammatory response is promoted; therefore, anti-S-IgG may be considered as additional activators of inflammation (Liu et al., 2019).

## NLR Family Pyrin Domain Containing 3 Inflammasome Is Activated following the Toll-Like Receptor Stimulation

SARS-CoV-2 may also bind the Toll-like receptors (TLRs) which are membrane receptors also found in immune cells, in T and B cells and in macrophages. Both the TLR 5 and TLR7, for example, are recognized by the sensing single-stranded RNA viruses (Kumar et al., 2009).

Once stimulated, TLR interacts with the intracellular Toll/IL-1 receptor (TIR) domain, inducing a pathway that may activate AP-1, NF-κB, and IRF3/7 that, in turn, promote the production of cytokines, chemokines, and type I interferons (IFNs) (Takeuchi & Akira, 2010).

In fact, since TLR5 modulation might play a protective role against respiratory infection, it has been hypothesized that the TLR5 immunomodulation might reduce cytokine storm production and restore the altered immune response following SARS-CoV-2 infection, even if this hypothesis has to be fully investigated (Chakraborty et al., 2020).

It has been recognized that SARS-CoV binds the TLR7, thus inducing the production of proinflammatory cytokines, such as TNF-α, IL-6, and IL-12 (Li et al., 2013), even if SARS-CoV effects following TLR7 activation still need to be investigated. A previous study showed that the use of a SARS coronavirus papain-like protease may inhibit the TLR7 pathway, thus reducing the activation of the transcription factors IRF3, NF-κB, and c-Jun and consequently IFN-α, IFN-β, TNF-α, IL-6, and IL-8 production (Li et al., 2016). SARS-CoV and SARS-CoV-2 show similarity; in fact, SARS-CoV-2 genome also contains fragments that could be recognized by TLR7/8, even if the affinity of SARS-CoV-2 on the TLR7 has not been confirmed yet (Moreno-Eutimio et al., 2020). TLR7 stimulation may induce IFN-regulated cytokines release and, interestingly, SARS-CoV-2, binding the TLR, may promote NLRP3 inflammasome activation (Conti et al., 2020), further inducing an inflammatory response and worsening the prognosis of COVID-19. The mechanism that describes how the TLR receptor may activate the NLRP3 inflammasome is related to the pattern recognition receptors (PRRs) (Kawai & Akira, 2010). Inflammasomes are activated by pathogen associated molecular patterns and damage associated molecular patterns, and once activated, on one hand they stimulate the production and the release of proinflammatory cytokines, and on the other hand they activate additional innate immune cells (Iwasaki and Medzhitov, 2015). TLRs activation following the interaction with a microbial agent causes the production of a supramolecular organizing center (SMOC), called myddosome, in cytoplasm. Myddosome stimulates the activation of the transcription factors involved in the production of proinflammatory cytokines: NF-κB and AP-1 (Kagan et al., 2014).

However, among the proinflammatory cytokines, the IL-1 pathway follows a different paradigm: PRRs activates IL-1 signaling, thus promoting the production of a SMOC called inflammasome (Martinon et al., 2002).

In particular, NLRP3 inflammasome is composed by NLRP3, apoptotic speck-containing protein adaptor, and procaspase-1. Procaspase-1 is cleaved into caspase-1 following NLRP3 activation and caspase-1 mediates the release of IL-1β and IL-18 (Martinon, 2010). SARS-Cov2 induces the production of IL-1β which is a mediator of lung inflammation, fever, and fibrosis, following TLR stimulation and inflammasome activation, thus contributing with an “additional via” to the cytokine storm during COVID-19.

## Therapeutic Approaches for the Treatment of Severe Acute Respiratory Syndrome Coronavirus-2 Cytokine Storm

Nowadays neither a specific treatment nor a vaccine has been found to manage SARS-CoV-2 infection; therefore the attention of the researchers worldwide is focused on the identification of an effective molecule to treat COVID-19.

Recent reports showed that more than 85% of patients with peculiar symptoms (fever, cough, and shortness of breath) received antiviral drugs, such as lopinavir and ritonavir tablets (400/100 mg twice a day per os) or remdesivir (once a day by intravenous injection) to ameliorate severe disease outcomes (Chen N. et al., 2020; Holshue et al., 2020; Li and De Clercq, 2020). These drugs have been introduced into COVID-19 treatment based on a virtual screening that identified lopinavir, an aspartate protease inhibitor, as a possible inhibitor of SARS-CoV-2 protease; ritonavir is usually administered with it since its inhibitory action on cytochrome P450 increases lopinavir plasma half-life. Unfortunately, more recently (Cao et al., 2020) a trial on 199 patients comparing the antiviral combination with standard treatment demonstrated no difference in the time to clinical improvement or mortality. On the other hand, remdesivir (GS-5734) has been approved by FDA for treating hospitalized COVID-19 patients on May 1, 2020. Remdesivir is an adenosine analog developed for Ebola and Marburg virus infection and its mechanism of action is based on the interaction with the viral RNA-dependent RNA polymerase thus reducing viral RNA production: in particular, the triphosphate form of remdesivir inhibits the viral RNA synthesis exploiting a specific mechanism of delayed chain termination (Saha et al., 2020a). Few weeks later a multicenter trial carried out in Hubei, China, on 158 patients treated with remdesivir versus 79 with standard care, failed to demonstrate a reduced time to clinical improvement or mortality, with a higher incidence of serious adverse events (Wang et al., 2020). In this scenario, another drug with strong anti-inflammatory effect and low interaction with plasma proteins and the CYP-P450 enzymes, baricitinib, has been proposed to be used in association with the antivirals lopinavir, ritonavir, and remdesivir (Stebbing et al., 2020). The rationale for baricitinib use relies on its mechanism of action; the drug is a Janus kinase (JAK) inhibitor, which blocks JAK-STAT pathway and the consequent release of cytokines. Usually, viral infection causes the release of type I IFN which interacting with its specific receptor induces JAK-STAT pathway activation causing a significant increase of circulating cytokines (Yu et al., 2017). Since the inflammatory response represents one of the major causes of lung damage and mortality, it was speculated that JAK inhibition may be useful to potentiate antivirals effect adding anti-inflammatory effects (Richardson et al., 2020). There are currently four clinical trials in Italy (NCT04320277), Spain (NCT04346147), and US (NCT04340232, NCT 04401579), evaluating the effect of baricitinib alone or in combination with antiviral drugs and the results are expected soon.

Other nucleoside analogs, such as favipiravir and ribavirin, are also used to treat viral infections of respiratory tract (Jordan et al., 2018) and might be effective for SARS-CoV-2. In fact, nucleoside analogs which derivate from adenine or guanine act on the RNA-dependent RNA polymerase, thus inhibiting viral RNA synthesis. Their efficacy remains uncertain because of their nonspecific action against SARS-CoV-2 and because of the possible appearance of side effects, such as anemia at high doses (Li and De Clercq, 2020). However, in a recent trial ribavirin was used in combination with lopinavir/ritonavir and interferon beta-1b with good results in reducing the time to negative swab (Hung et al., 2020), while the combinations of i) favipiravir and IFN-α and ii) favipiravir and baloxavir marboxil are currently under investigation in patients with COVID-19. It is important to note that the use of antivirals represents a nonspecific approach to treat SARS-CoV-2 since coronaviruses have different proteases: a possible new therapeutic strategy might be to synthesize a specific (i.e., a cysteine protease inhibitor) or a wide spectrum inhibitor.

At the present moment, also other therapeutic approaches are controversial; for many weeks chloroquine and hydroxychloroquine, widely used for the treatment and the prevention of malaria, have been also administered in patients affected by COVID-19. As for SARS-CoV and H5N1, chloroquine inhibits SARS-CoV-2 entry into the cells by glycosylating ACE2 receptors (Vincent et al., 2005); clinical trials showed that it may reduce the number of hospitalizations, ameliorating patient outcomes (Savarino et al., 2006; Yan et al., 2013). Also WHO included these antimalarial as treatments in the SOLIDARITY trial (NCT04330690), but very recently a multinational registry analysis on 96,032 patients around the globe failed to demonstrate a real benefit of hydroxychloroquine or chloroquine used alone or with a macrolide on the final outcome (Mehra et al., 2020). Among the other antiviral strategies, umifenovir (Arbidol) is a new antiviral option approved in China for the management of COVID-19: it prevents virus entry by binding to hemagglutinin (Aktas et al., 2020). However, clinical studies on umifenovir showed that the administration of umifenovir alone was ineffective, whereas the combination with other antivirals might ameliorate viral clearance and improve lung damage (Chen et al., 2020; Song et al., 2020).

Besides ACE2 binding, SARS-CoV-2 exploits the serine protease TMPRSS2 for S-protein priming; therefore, the use of a TMPRSS2 inhibitor, such as camostat mesylate, might inhibit SARS-CoV-2 infection (Hoffmann et al., 2020); however there are no results available from the clinical trials that have been registered in Israel (NCT04355052), Denmark (NCT04321096), and Kentucky (NCT04374019) although they might have promising results since TMPRSS2 inhibitors may directly act on SARS-CoV-2 and may also have anti-inflammatory effect, reducing the transcription of proinflammatory genes.

Although the treatments described so far are based on the reduction of viral spread, other molecules exploit their immunomodulatory and anti-inflammatory effects against SARS-CoV-2 reducing cytokines. The cytokine storm contributes to the hyperactivation of T cells which further increases proinflammatory cytokines levels, worsening the disease related to SARS-CoV-2 infection, inducing T cell exhaustion, apoptosis, acute lung injury, and eventually acute respiratory distress syndrome (Channappanavar et al., 2016). In this context, IL-6 and TNF-α are the main proinflammatory molecules responsible for the cytokine storm that play a pivotal role in the viral infection and in associated lung damage, as demonstrated in COVID-19 patients with severe symptoms and poor prognosis (Chen X. et al., 2020; Fu et al., 2020). Also tocilizumab, a humanized monoclonal antibody, has been proposed and in some cases successfully used to halt the cytokine storm during COVID-19. Tocilizumab is used for the management of autoimmune diseases as rheumatoid arthritis that recognize a strong cytokine activation; in fact by binding the IL-6 receptor the drug is able to inhibit both classical and trans IL-6 downstream pathway (Saha et al., 2020b). In particular, a recent study showed that tocilizumab was able to reduce symptoms and improve the outcomes of patients with a severe SARS-CoV-2 infection (Dholaria et al., 2019; Xu et al., 2020). Tocilizumab has been included in the Chinese and Italian guidelines and may only be used following the acute phase of SARS-CoV-2 infection, 7 days following the beginning of symptoms, or in patients without fever and high levels of IL-6 (NHC, 2020). Among the proinflammatory cytokines, IL-1 also plays an important role in exacerbating inflammation during COVID-19 in fact, SARS-CoV-2 induces pyroptosis with the consequent release of IL-1β (Jiang et al., 2019). Therefore, targeting IL-1 receptor may be useful to reduce IL-1 release and cytokine storm: anakinra, for example, is a recombinant human IL-1 receptor antagonist, approved for rheumatoid arthritis, which improved the survival rate of patients with SARS-Cov-2 infection (Shakoory et al., 2016). In a recent trial on 52 COVID-19 treated with anakinra a significant reduction of mortality was observed, along with a significant decrease in the need for mechanical invasive ventilation, with no evident adverse events (Huet et al., 2020).

Besides these drugs there is a lot of literature revealing that the use of natural products was recommended for the prevention and treatment of acute respiratory syndrome (Liu et al., 2004) and four Chinese herbal medicines were suggested in the prevention program during the H1N1 influenza (National Administration of Traditional Chinese Medicine, 2009; Cragg and Newman, 2013). The application of the traditional Chinese medicine for the treatment of SARS-CoV-2 is currently based on the treatments used for SARS-CoV, thanks to their similarities (Gong et al., 2008; Nguyen et al., 2012). A retrospective observational study carried out in 293 patients affected by COVID-19 showed that the prescription of common herbs used in folk medicine may be considered as an integrative approach for COVID-19 management, since some popularly used drugs, such as moxifloxacin, oseltamivir, and Arbidol, are not always effective in reducing severe symptoms (Shu et al., 2020). However, since COVID-19 is a complex clinical disease that is often complicated by severe disorders, as clotting activation, rapid degeneration of parenchyma, and organ failure, additional studies will be requested to confirm the efficacy of traditional medicine approach alone or in combination with other drugs.

As previously described, SARS-CoV-2 infects target cells through ACE2 binding; therefore, targeting ACE2 with natural compounds might prevent SARS-CoV-2 infection. For instance, baicalin is a natural component extracted from *Scutellaria baicalensis* and its antiviral and anti-inflammatory activity have been demonstrated also during coronavirus infection. As a matter of fact, this natural molecule was proven effective against H1N1 virus and respiratory syncytial virus infection, and last but not least, a recent study demonstrated that baicalin significantly inhibited SARS-CoV S-protein binding to ACE2 (Zhang et al., 2011; Deng et al., 2012; Cheng et al., 2014). Moreover, it is worthy of interest to consider that baicalin exerts potent anti-inflammatory effects and significantly may reduce cytokines production (Li et al., 2000). Therefore, baicalin, as well as other natural products, might be considered as potential therapeutic approaches for the treatment of SARS-CoV-2 infection and the associated cytokine storm production, thus reducing the proinflammatory cytokines release and avoiding the progression of COVID-19 to a severe disease that might increase the rate of mortality. Natural products might be useful to prevent and alleviate SARS-CoV-2 infection in the near future, but *in vivo* and later clinical studies will be needed to investigate their possible antiviral and anti-inflammatory effects.

## Conclusion

Although the whole world is focused on finding a cure for COVID-19 symptoms and a vaccine for SARS-CoV-2, it seems that a way out is still far; in these times collaboration and communication among governments and scientists of different countries is important and might be the only way to provide the necessary solution for this crisis (Chakraborty et al., 2020).

So far, no specific antiviral therapy or vaccine has been identified for the treatment or prevention of SARS-CoV-2 infection. Potential therapeutic approaches here discussed are focused on blocking the virus from infecting cells or on limiting the exaggerated inflammatory response related to COVID-19 to prevent severe and fatal lung injury. The numerous clinical trials that have been registered during the emergency in the various countries were not extensively reviewed by the Institutional Review Board and may suffer from important biases, as the lack of appropriate control groups, which makes it even harder to obtain a proper interpretation of the results. Nonetheless, it is important to advise that the whole scientific community has learned lots of important issues in trying to find a cure for this disease and in some cases the best clinical results have been obtained empirically using a combination of different drugs or natural products, suggesting that probably this could be the way to fight this multiorgan disease.

## Author Contributions

FM and NI wrote the paper; AB made critical revision.

## Conflict of Interest

The authors declare that the research was conducted in the absence of any commercial or financial relationships that could be construed as a potential conflict of interest.
